# A systematic evaluation of miRNA:mRNA interactions involved in the migration and invasion of breast cancer cells

**DOI:** 10.1186/1479-5876-11-57

**Published:** 2013-03-05

**Authors:** Daya Luo, James M Wilson, Nikki Harvel, Jimei Liu, Lirong Pei, Shuang Huang, LesleyAnn Hawthorn, Huidong Shi

**Affiliations:** 1 Medical College of Nanchang University, Nanchang, Jiangxi, PR China; 2 GHSU Cancer Center, Georgia Health Sciences University, Augusta, GA, USA; 3 Department of Biochemistry and Molecular Biology, Georgia Health Sciences University, Augusta, GA, USA; 4 Department of Pathology, Georgia Health Sciences University, Augusta, GA, USA

**Keywords:** Breast cancer, MicroRNA target genes, Migration, Invasion

## Abstract

In this study we performed a systematic evaluation of functional miRNA-mRNA interactions associated with the invasiveness of breast cancer cells using a combination of integrated miRNA and mRNA expression profiling, bioinformatics prediction, and functional assays. Analysis of the miRNA expression identified 11 miRNAs that were differentially expressed, including 7 down-regulated (miR-200c, miR-205, miR-203, miR-141, miR-34a, miR-183, and miR-375) and 4 up-regulated miRNAs (miR-146a, miR-138, miR-125b1 and miR-100), in invasive cell lines when compared to normal and less invasive cell lines. Transfection of miR-200c, miR-205, and miR-375 mimics into MDA-MB-231 cells led to the inhibition of *in vitro* cell migration and invasion. The integrated analysis of miRNA and mRNA expression identified 35 known and novel target genes of miR-200c, miR-205, and mir-375, including *CFL2*, *LAMC1*, *TIMP2*, *ZEB1*, *CDH11*, *PRKCA*, *PTPRJ*, *PTPRM*, *LDHB*, and *SEC23A*. Surprisingly, the majority of these genes (27 genes) were target genes of miR-200c, suggesting that miR-200c plays a pivotal role in regulating the invasiveness of breast cancer cells. We characterized one of the target genes of miR-200c, *CFL2*, and demonstrated that *CFL2* is overexpressed in aggressive breast cancer cell lines and can be significantly down-regulated by exogenous miR-200c. Tissue microarray analysis further revealed that CFL2 expression in primary breast cancer tissue correlated with tumor grade. The results obtained from this study may improve our understanding of the role of these candidate miRNAs and their target genes in relation to breast cancer invasiveness and ultimately lead to the identification of novel biomarkers associated with prognosis.

## Introduction

MicroRNAs (miRNAs) are a class of small non-coding RNA molecules 19-24 nucleotides in length that suppress gene expression post-transcriptionally by base-pairing with the 3^′^-untranslated regions (3^′^-UTRs) of target mRNA [1]. Recent studies have shown that miRNAs are involved in multiple processes of cancer progression including cancer cell proliferation and metastasis [2]. Large scale profiling approaches have revealed that miRNAs are globally down-regulated in breast cancer [3]. The same study identified a set of miRNAs as being differentially expressed in breast tumors and showed that the miRNA profile could be used to distinguish between breast cancer and normal breast tissue [3]. Studies have also revealed the correlation between down-regulation of certain miRNAs and clinicopathological features such as ER/PR positivity, tumor size, lymph node status, and metastasis status [3,4]. It was reported that the level of miR-31 was down-regulated [5], while the level of miR-10b was up-regulated in metastatic breast tumors [6]. With an experimental murine model, two reports have shown that tumors can be effectively suppressed by either silencing pro-cancer miRNA (miR-10b) [7] or expressing anti-cancer miRNA (miR-26a) [8]. Taken together, these findings clearly demonstrate that miRNAs play a critical role in breast cancer development and progression.

Metastasis is the main cause of breast cancer mortality and involves multiple complicated processes [9,10]. The ability of mammary tumor cells to invade and destroy neighboring tissues and organs, as well as migrate to other parts of the body, is crucial to the metastatic process [9,10]. It has been increasingly recognized that miRNAs regulate cell migration and invasion and play an important role in the invasiveness of breast cancer cells [11,12]; however, a systematic investigation of how miRNAs affect the invasive behavior of breast cancer cell lines has not been conducted. In this study, we performed an integrated analysis of miRNA and mRNA expression profiles in 12 breast cancer cell lines and identified a group of miRNA that are differentially expressed in invasive breast cancer cell lines when compared to less-invasive cell lines. We identified 35 functional target genes of three significantly down-regulated miRNAs in invasive cell lines, namely miR-200c, miR-205, and miR-375. Extensive validation studies were performed to confirm the functional interaction of the three miRNAs and their target genes. Finally, we characterized one of the target genes of miR-200c, *CFL2*, and demonstrated that *CFL2* is overexpressed in invasive breast cancer cell lines and regulated by miR-200c. Tissue microarray analysis (TMA) further demonstrated that CFL2 expression in primary breast cancer tissue was positively correlated with tumor grades.

## Materials and methods

### Tissue culture and RNA isolation

Breast cancer cell lines BT474, MDA-MB-468, T47D, ZR-75-1, MCF7, SK-BR3, MDA-MB-231, HS578T, BT549, SUM159, and HeLa cell line were cultured in DMEM media supplemented with 10% fetal bovine serum (FBS). Immortalized breast epithelium cell lines MCF10A and MCF12A were cultured in DMEM/F12 supplemented with 5% horse serum, 20 ng/mL EGF, 10 μg/mL insulin, 100 ng/ml cholera toxin, and 500 ng/ml hydrocortisone (all from Sigma Aldrich, St Louis, MO). Total RNA was extracted using the QIAzol™ Lysis reagent (Qiagen, Valencia, CA). Small molecular weight RNA was extracted using the mirVana™ miRNA Isolation Kit (Invitrogen, Carlsbad, CA) per manufacturer’s protocol.

### Transfection

miR-200c, miR-205, miR-375 mimic or scrambled negative control (Ambion, Austin, TX) at a concentration of 50 nM were incubated with Lipofectamine 2000 (Invitrogen) in culture medium before addition to cells according to the manufacturer's protocol. *CFL2* siRNA and scramble control siRNA were purchased from Dharmacon (Lafayette CO.) and used at a concentration of 30 nM as described above.

### microRNA expression profiling

The GeneChip miRNA 1.0 array (Affymetrix, Santa Clara, CA) was used for the miRNA expression profiling in breast cancer cell lines. One μg of small RNA from each sample was labeled with biotin using the FlashTag Biotin RNA Labeling Kit (Genisphere, Hatfield, PA). Array hybridization, washing, and scanning of the slides were carried out according to Affymetrix's recommendations. Data was extracted from the images, quantile-normalized, summarized (median polish), and log_2_-transformed with the miRNA QC software from Affymetrix. Partek Genomic Suites (Partek, St. Louis, MO) was used to analyze the array results, and TargetScan6.2 ( http://www.targetscan.org/) was used to predict miRNA-mRNA pairs. All microarray data has been submitted to NCBI Gene Expression Omnibus ( http://ncbi.nlm.nih.gov/geo/) under accession number GSE40059.

### mRNA expression profiling

The GeneChip® Human Genome U133 Plus 2.0 Array (Affymetrix) was used for the mRNA expression profiling in 12 breast cancer cell lines. Biotinylated cRNA was synthesized from total RNA using the Affymetrix 3^′^ IVT Express Kit according to manufacturer’s protocols. The GeneChip® Human Gene 1.0 ST Array (Affymetrix) was used for the mRNA expression analysis in the miRNA mimic transfected MDA-MB-231 cells. The cRNA was synthesized using Ambion WT Expression Kit and labeled using Affymetix GeneChip WT Terminal Labeling Kit. Array hybridization, washing, and scanning of the slides were carried out according to Affymetrix's protocols. The gene expression data was analyzed using Partek Genomic Suites 6.5. The Ingenuity Pathway analysis (IPA) was used to identify functional groups and molecular networks from the microarray data sets generated in the miRNA mimic transfected MDA-MB-231 cells.

### qRT-PCR analysis of miRNA expression

One μg of small RNA was used for reverse transcription with the RT^2^ miRNA First Strand Kit (SA Biosciences, Frederick, MD). Quantitative RT-PCR was carried out using a Light Cycler 480 II instrument (Roche, Indianapolis, IN). The PCR primers for U6, miR-200c, miR-205, miR-375, and miR-146a were purchased from SABiosciences. RT^2^ SYBR Green Master Mixes (SA Biosciences) were used in the real time PCR reaction according to the manufacturer’s suggested protocols. The relative gene expression was calculated using the equation 2^-ΔCt^, where ΔCt = Ct (miRNA) − Ct (U6).

### qRT-PCR analysis of mRNA expression

Two μg of the total RNA was reverse-transcribed using the High Capacity cDNA Reverse Transcription Kit (Applied Biosystems, Foster City, CA). All PCR reactions were carried out as described above. The primer sequences used for RT-PCR can be found in Additional file 1: Table S1. Each sample was run in duplicate. Fold change in gene expression was calculated using ΔΔCt method.

### Transwell migration and invasion assay

miRNA mimic or siRNA treated and control cells were starved in serum-free medium for 2 hours, detached, and then re-suspended in medium with 2.5% fetal bovine serum at a density of 4 × 10^5^ cells/mL. For the migration assay, 500 μL of the cell suspension was added to the upper chamber of the transwell inserts (BD Biosciences, Sparks, MD). 750 μL of medium containing 10% fetal bovine serum was added into the bottom of a 24 well plate to act as a chemoattractant. After an 8-hour migration period, non-migratory cells in the upper chamber were removed with cotton swabs, and the cells on the lower surface of the inserts were fixed and stained using DIFF-QUICK (IMEB Inc, San Marcos, CA). The number of migratory cells was calculated by counting five different fields under a phase-contrast microscope in three independent inserts. Invasion assays were done in a similar manner as the migration assays described above, except that the inserts were pre-coated with Matrigel (BD Biosciences). The cells were allowed to invade for 24 hours before proceeding with fixation and staining.

### Luciferase reporter assay

The 3^′^-UTR of *CDH11*, *CFL2*, *SEC23A*, *ZEB-1*, *PTPRM*, and *LDHB* were generated by PCR using DNA isolated from HeLa cells. The PCR fragments were subcloned into the pmirGLO dual-luciferase reporter vector (Promega, Madison, WI, USA). The primers used for 3^′^-UTR amplification can be found in Additional file 2: Table S2. The reporter gene constructs were cotransfected into HeLa cells containing a miR mimic control or miR-200c/205/375 mimic for 48 hours. The dual luciferase system (Promega) was used to measure luciferase activity per manufacturer’s protocol. Normalized firefly luciferase activity (firefly luciferase activity/Renilla luciferase activity) was used to compare each respective sample to the control. For each transfection, luciferase activity was averaged from three replicates.

### F-actin staining

Cells were fixed in 3.7% formaldehyde solution and extracted with a solution of 0.1% Triton X-100 in PBS for 5 minutes. The cells were then washed three times with PBS and stained using a rhodamine phalloidin (Invitrogen) solution for 20 minutes at room temperature. The cells were washed three times with PBS and mounted in a Mounting Medium for Fluorescence (Vector Laboratories, Inc. Burlingame, CA).

### Immunohistochemistry

CFL2 levels in breast tumors and normal breast tissues were evaluated by IHC using anti-CFL2 polyclonal antibody (1:250 dilution, sc-32160, Santa Cruz Biotechnology, Santa Cruz, CA) on commercial tissue arrays (Shanghai Outdo Biotech Co., Shanghai) as previously described [13]. The array contained 5 normal breast tissues and 211 breast tumor specimens. Staining intensity of each sample was given a modified histochemical score (MH-score) that considers both the intensity and the percentage of cells stained at each intensity [14]. The intensity of each grade is the average of MH-score of all samples in that grade. Clinicopathological data of the 211 tumors used in TMA is provided in Additional file 3: Table S3.

### Statistical analysis

Each experiment was repeated at least in triplicate. Numerical data are presented as mean ± s.d. Student’s *t*-test was used to analyze the differences between two samples; differences were considered statistically significant at p < 0.05. One-way ANOVA was performed in SPSS 17.0 (SPSS Inc. Chicago, IL) to analyze the association of CFL2 and tumor grades.

## Results

### Differential miRNA expression between invasive and less-invasive breast cancer cell lines

**Figure 1:**
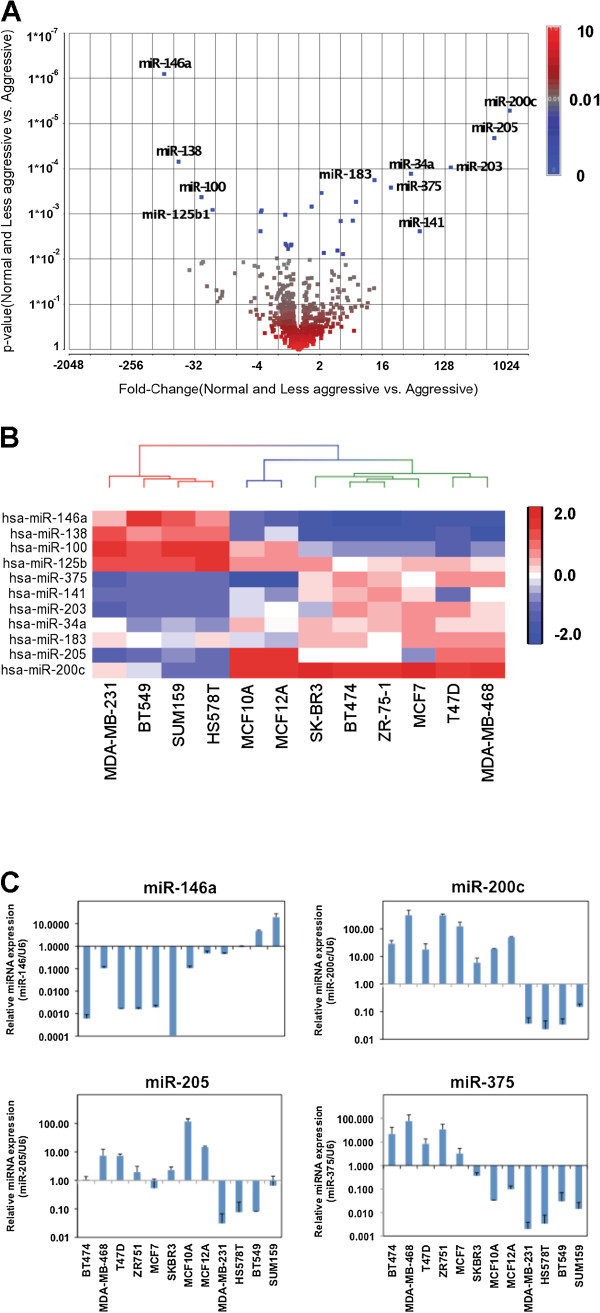
**Differential miRNA expression in 12 breast cancer cell lines.** (**A**) A volcano plot shows that 19 out of 847 miRNAs were differentially expressed (p < 0.01 and fold-change >2). 11 miRNAs (labeled) have an average fold-change above 10 between aggressive and less-aggressive groups. (**B**) Heatmap representing the expression values of 11 miRNAs that are differentially expressed between aggressive and less aggressive groups. (**C**) qRT-PCR confirmation of the miRNA array results for miR-200c, miR-205, miR-375, and miR-146a in the 12 breast cell lines.

We first performed miRNA expression profiling of 847 known human miRNAs in 6 less-invasive breast cancer cell lines (BT474, MDA-MB-468, T47D, ZR-75-1, MCF7, SK-BR3), 4 invasive cell lines (MDA-MB-231, HS578T, BT549, SUM159), and two non-tumorigenic breast epithelial cell lines (MCF10A and MCF12A). One-way ANOVA analysis was used to identify differentially expressed miRNAs between each group of cell lines. Using stringent criteria (p < 0.01 and fold-change >2), 19 of the 847 miRNA genes were found to be differentially expressed with statistical significance between the two groups of cell lines (Figure 1A). 11 of the 19 miRNAs have an average fold-change above 10 between the invasive and less-invasive groups. Among the 11 most differentially expressed miRNAs, miR-141, miR-183, miR-200c, miR-205, miR-203, miR-34a, and miR-375 were down-regulated in invasive cell lines when compared to the normal and less invasive lines; conversely, miR-100, miR-125b1, miR-138, and miR-146a were found to be up-regulated (Figure 1A). Supervised cluster analysis using the miRNA expression values demonstrated that the three groups of breast cancer cell lines can be clearly separated by the expression profiles of the 11 miRNAs (Figure 1B). Quantitative RT-PCR (qRT-PCR) analysis of individual miRNAs confirmed that miR-200c, miR-205, miR-375, and miR-146a were indeed differentially expressed between the two groups of breast cancer cell lines (Figure 1C). In particular, miR-200c, miR-205, and miR-375 were found to be down-regulated more than 100 fold on average.

### Transient transfection of miR-200c, -205, and -375 inhibits cell migration and invasion

**Figure 2:**
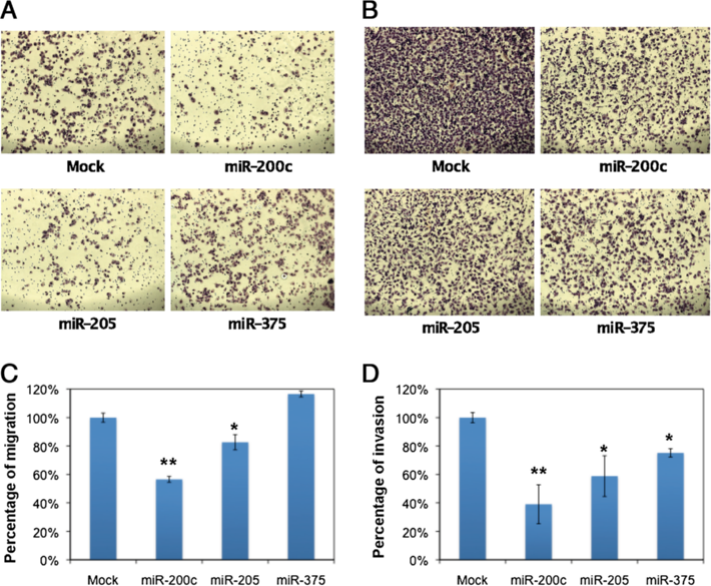
**Migration and invasion assays for miR-200c, miR-205, miR-375 mimic transduced MDA-MB-231 cells.** (**A**) Migration assay for miRNA-transduced MDA-MB-231 cells (100×). (**B**) Invasion assay for miRNA-transduced MDA-MB-231 cells (100×). (**C**) Bar graph representing the percentage of reduction in cell migration after the respective miRNA transfection. (**D**) Bar graph representing the percentage of reduction in cell invasion after the respective miRNA transfection. *: Student’s *T*-test, p < 0.05; **: Student’s *T*-test <0.01.

Because miR-200c, miR-205, and miR-375 have been previously reported to affect cell migration or invasion [15,16], and were all found to be down-regulated in the invasive cell lines, we investigated the effects of transient transfection of these three miRNAs on breast cancer cell migration and invasion. As shown in Figure 2, transfection of miR-200c into the invasive breast cancer cell line MDA-MB-231 had the greatest impact on both cell migration and invasion, both of which decreased by approximately 50%. Transfection of miR-205 into the MDA-MB-231 cells was less effective than miR-200c, but cell migration and invasion were still reduced by about 20%. miR-375 was found to play the opposite role in regulating migration and invasion in MDA-MB-231 cells as the migration and invasion changed in the opposite direction (Figures 2C-D). The results from these experiments confirmed that these three miRNAs were capable of regulating cell migration and invasion and therefore ultimately affect the invasiveness of breast cancer cells.

### Differential mRNA expression between invasive and less-invasive breast cancer cell lines

**Figure 3:**
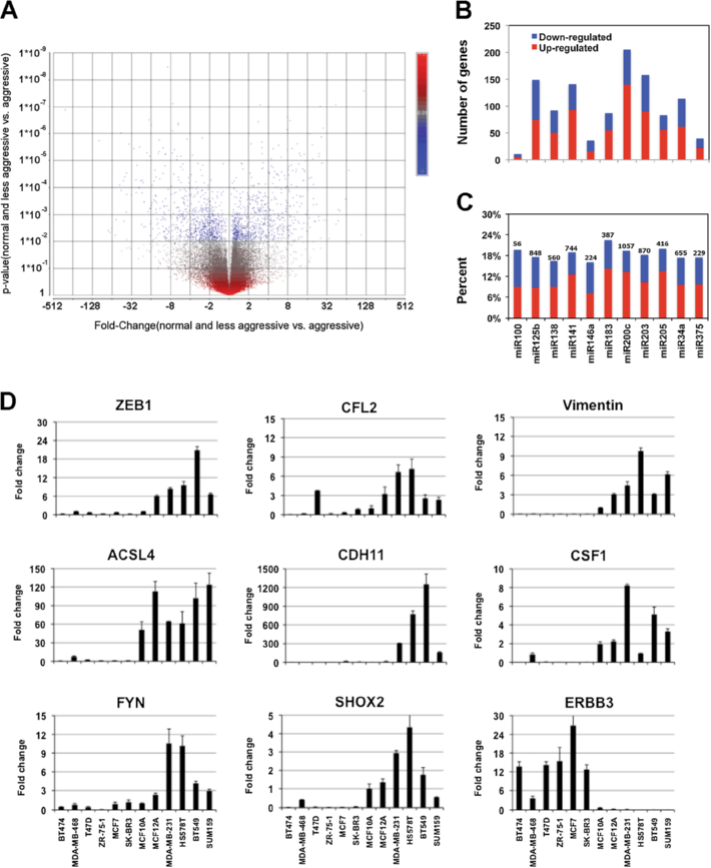
**Differential gene expression in 12 breast cancer cell lines.** (**A**) A volcano plot shows that 2412 genes were differentially expressed (p < 0.05 and fold-change >1.5) between normal and less aggressive cell lines vs. aggressive cell lines. (**B**) The number of predicted miRNA target genes that were up or down-regulated in aggressive breast cancer cell lines. (**C**) The percentage of predicted miRNA target genes that were up and down-regulated in aggressive breast cancer cell lines. The number above each bar represents the number of predicted target genes of each miRNA by TargetScan 6.2. (**D**) Confirmation of 9 miRNA target genes using qRT-PCR in 12 breast cancer cell lines. Fold change was calculated using ΔΔCt method. MCF10A was used as the control and therefore the fold change for MCF10A is 1 in all plots.

We performed gene expression microarray analysis using the same set of breast cancer cell lines with the aim of identifying differentially expressed genes that might be target gene candidates for the differentially expressed miRNA. The Affymetrix GeneChip analysis showed that 2412 genes were differentially expressed (p <0.05 and fold-change >1.5) between normal and less-invasive cell lines vs. invasive cell lines (Figure 3A). Among the 2412 genes, 1291 genes were up-regulated and 1121 genes were down-regulated in invasive breast cancer cell lines as compared to normal and less invasive cell lines. The lists of up and down-regulated genes are provided in Additional file 4: Table S4 and Additional file 5: Table S5. We compared the lists of up or down regulated genes in the invasive cell lines with the lists of the predicted targeted genes of the above-mentioned 11 miRNAs, respectively. The numbers of predicted miRNA target genes that were differentially expressed were quite variable (Figure 3B). The predicted target genes of miR-200c, miR203, miR125b, and miR-141 were the most abundant, while those of miR-100, miR-146a, and miR-375 were the least abundant among the up or down-regulated genes in invasive cell line. However, the observed difference was mainly due to the difference in the number of predicted target genes for each miRNA. For instance, TargetScan 6.2 predicted 1057 target genes for miR-200c, but only 56 target genes for miR-100. When we compared the percentage of the predicted miRNA target genes that were up or down regulated in invasive cell lines, the differences became smaller; only 15%-23% of the predicted target genes of each miRNA were differentially expressed between less-invasive cell lines vs. invasive cell lines (Figure 3C). Since the function of miRNA is to repress gene expression, we expected to see a negative correlation between the expression of miRNAs and the expression of their target genes. This seems to be true for miR-200c, miR-205, and miR-141 (all down-regulated in invasive cell lines) as twice as many predicted target genes were up-regulated in invasive cell lines than were down-regulated. However, we did not observe an obvious negative correlation in the other miRNAs; in particular, we did not see the negative correlation in the miRNAs that were up-regulated in invasive cell lines such as miR-100, miR-138, and miR-146a. We randomly selected 9 candidate genes with variable fold change values and performed qRT-PCR analysis. qRT-PCR analysis confirmed the gene expression array results for all 7 miRNA target genes including *ZEB1*, *CFL2*, *ACSL4*, *CDH11*, *CSF1*, *FYN*, and *SHOX2* (Figure 3D). *Vimentin*, one of the most differentially expressed genes, was used as the control. *ERBB3*, which is a predicted target of miR-205, was shown to be down-regulated in invasive cell lines as determined by array analysis. The qRT-PCR results also confirmed the results.

### Exogenous miR-200c, miR-205, and miR-375 mimics cause significant gene expression changes

**Figure 4:**
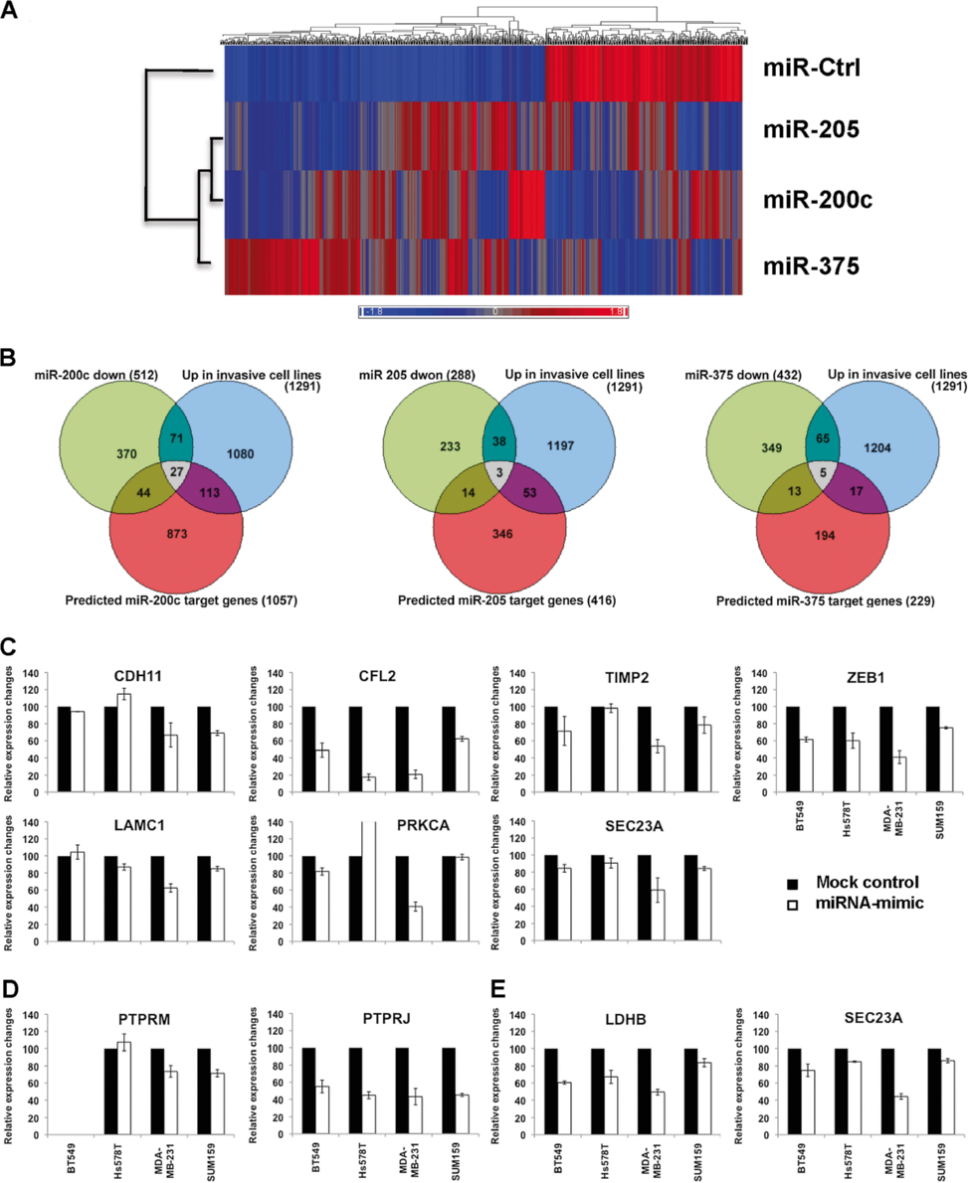
**Integrative analysis of mRNA and miRNA expression in breast cancer cells.** (**A**) Heatmap representing the differentially expressed genes in miR-200c, miR-205, and miR-375 mimic transfected MDA-MB-231 cells. (**B)** Venn diagrams showing the intersection between: green circle, mRNA transcripts displaying at least a 1.4-fold decrease in expression in miR-200c, miR-205, and miR-375 mimic transfected MDA-MB-231 cells; blue circle, mRNA transcripts (p < 0.05 and fold change >1.5) that showed an increased expression in aggressive vs. normal and less aggressive cell lines. red circle, likely mRNA targets for miR-200c, miR-205, and miR-375 predicted using TargetScan6.2. The grey area indicates the intersection of all 3 circles. (**C**-**E**) qRT-PCR analysis of 11 candidate target genes of miR-200c (**C**), miR-205 (**D**), and miR-375 (**E**) in miR-200c, miR-205, and miR-375 transduced breast cancer cell lines, BT549, HS578T, MDA-MB-231 and SUM159, respectively.

In order to identify the potential direct targets of miR-200c, miR-205, and miR-375, each miRNA was individually transfected into the MDA-MB-231 cell line. An Affymetrix 1.0 Gene ST array analysis was then performed using the transfected MDA-MB-231 cells to identify differentially expressed genes caused by each individual miRNA mimic. Cluster analysis of the microarray data generated from the miRNA transduced cell line showed that a large number of genes were affected (Figure 4A). IPA anlaysis revealed that there was a significant enrichment of genes involved in cellular movement, cell-to-cell signaling and interaction, cellular growth and proliferation, inflammatory response, and cancer (Additional file 6: Figure S1). Analysis of the differentially expressed genes in the miRNA mimic transfected cells performed using the Ingenuity Knowledge Base generated several molecular networks for each miRNA. One of the networks identified in the miR-200c mimic transfected cells was found to be most interesting (Additional file 6: Figure S2A). The network analysis mapped CDH1 (E-cadherin) to the core of this network, acting as a hub connected by several neighborhood genes that play important roles in cell migration and invasion. Overall, we identified 512, 287, and 432 down-regulated genes (fold change >1.4) in miR-200c, miR-205, and miR-375 mimic transfected MDA-MB-231 cells, respectively (Additional file 7: Tables S6, Additional file 8: Table S7 and Additional file 9: Table S8). Only 28 genes were down-regulated in all three mimic-transfected cell lines.

### Identification of the candidate miRNA target genes through integrative analysis

We compared the list of genes down-regulated in miRNA-mimic transfected MDA-MB-231 cells with the list of genes up-regulated in invasive breast cancer cell lines, as well as the list of potential target genes for each microRNA as predicted by TargetScan 6.2. The Venn diagrams in Figure 4B show the intersections among the three lists. The list of genes generated by the Venn diagram analysis is provided in Table 1. Among the 35 genes, 27 genes were identified as miR-200c target genes, only three genes were miR-205 targets, and six genes were miR-375 targets. We selected 10 genes for the confirmation study based on their potential roles in breast cancer and *ZEB-1* was used as a control. The qRT-PCR analysis demonstrated that the expression of *CDH11*, *CFL2*, *LAMC1*, *PRKCA*, *SEC23A*, *TIMP2*, *ZEB-1* (miR-200c target genes), *PTPRM*, *PTPRJ* (miR-205 target genes), *SEC23A* and *LDHB* (for miR-375 target genes) decreased by more than 30% in the MDA-MB-231 cells transfected with each individual miRNA mimic (Figure 4C-E), respectively. *SEC23A* was found to be down-regulated by both miR-200c and miR-375 mimics. Since we only performed array analysis using MDA-MB-231 cells, we tested to see if the miRNA mimics could down-regulate their target genes in three other invasive cell lines. *CFL2* and *ZEB1* (miR-200c target genes), *PTPRJ* (miR-205 target gene), and *LDHB* (miR-375 target gene) were consistently down-regulated in all four cell lines transfected with the corresponding miRNA mimics; however, the remainder of the genes displayed variable results (Figures 4C-E).

**Table 1:** List of target genes of miR-200c, miR-205, and miR-375 identified in breast cancer cell lines

**miR**	**Target Gene**	**Accession No.**	**Name**
**miR-200c**	*ANKH*	NM_054027	ankylosis, progressive homolog (mouse)
	*AP1S2*	NM_003916	adaptor-related protein complex 1, sigma 2 subunit
	*CDH11*	NM_001797	cadherin 11
	*CFL2*	NM_021914	cofilin 2 (muscle)
	*CRTAP*	NM_006371	cartilage associated protein
	*DPY19L1*	NM_015283	dpy-19-like 1 (C. elegans)
	*DENND5B*	NM_144973	DENN/MADD domain containing 5B
	*EMP1*	NM_001423	epithelial membrane protein 1
	*FAM46C*	NM_017709	family with sequence similarity 46, member C
	*FN1*	NM_212474	fibronectin 1
	*FOXF2*	NM_001452	forkhead box F2
	*FSCN1*	NM_003088	fascin homolog 1, actin-bundling protein (Strongylocentrotus purpuratus)
	*KANK2*	NM_001136191	KN motif and ankyrin repeat domains 2
	*LAMC1*	NM_002293	laminin, gamma 1 (formerly LAMB2)
	*LHFP*	NM_005780	lipoma HMGIC fusion partner
	*LOX*	NM_001178102	lysyl oxidase
	*MCFD2*	NM_139279	multiple coagulation factor deficiency 2
	*PAG1*	NM_018440	phosphoprotein associated with glycosphingolipid microdomains 1microdomains 1
	*PDE7B*	NM_018945	phosphodiesterase 7B
	*PPP1R18*	NM_133471	protein phosphatase 1, regulatory subunit 18
	*PRKCA*	NM_002737	protein kinase C, alpha
	*RGL1*	NM_015149	ral guanine nucleotide dissociation stimulator-like 1
	*SEC23A*	NM_006364	Sec23 homolog A (S. cerevisiae)
	*SPRED1*	NM_006931	sprouty-related, EVH1 domain containing 1
	*TIMP2*	NM_003255	TIMP metallopeptidase inhibitor 2
	*ZEB1*	NM_030751	zinc finger E-box binding homeobox 1
	*ZEB2*	NM_014795	zinc finger E-box binding homeobox 2
**miR-205**	*CALU*	NM_001219	calumenin
	*PTPRJ*	NM_002843	protein tyrosine phosphatase, receptor type, J
	*PTPRM*	NM_001105244	protein tyrosine phosphatase, receptor type, M
**miR-375**	*DIP2C*	NM_014974	DIP2 disco-interacting protein 2 homolog C
	*KIAA1199*	NM_018689	KIAA1199, colon cancer secreted protein 1
	*LDHB*	NM_002300	lactate dehydrogenase B
	*QKI*	NM_006775	quaking homolog, KH domain RNA binding
	*SEC23A*	NM_006364	Sec23 homolog A (S. cerevisiae)
	*SLC7A11*	NM_014331	solute carrier family 7

**Figure 5:**
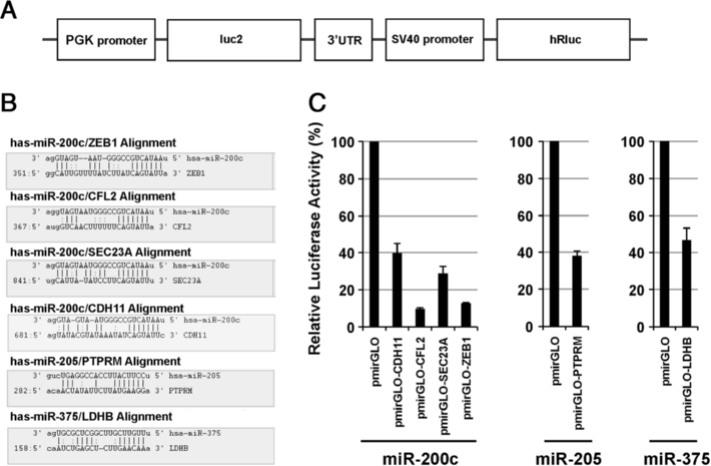
**3**^′^**-UTR reporter assays confirming the interaction of individual miRNA with the 3**^′^**-UTR of candidate target genes.** (**A**) Schema of the pmirGLO dual luciferase vector carrying the 3^′^-UTR regions of 6 selected genes. The entire 3^′^-UTR of 6 genes: *CDH11*, *CFL2*, *SEC23A*, *ZEB-1*, *PTPRM*, and *LDHB* were cloned into the pmirGLO dual luciferase vector. (**B**) The alignments of miRNA and their predicted target genes. (**C**) Percent relative luciferase activity 48 hours post-transfection with the indicated reporter vector.

In order to determine whether the candidate miRNA targeted genes are regulated by each miRNA through direct 3^′^-UTR interaction, we cloned the 3^′^-UTR of *CDH11*, *CFL2*, *SEC23A*, *ZEB-1* (miR-200c target), *PTPRM* (miR-205 target), and *LDHB* (miR-275 target) into the reporter plasmid pmirGLO to generate gene-specific 3^′^-UTR luciferase reporter vectors. These plasmids and the vector control plasmid were transiently co-transfected into Hela cells with the corresponding miRNA mimics. After 48 hours, a dual-luciferase reporter assay system was used to measure luciferase expression. Overexpression of each individual microRNA resulted in a significant decrease of luciferase activities (Figure 5). These results further confirmed that these genes were the true targets of the corresponding miRNAs.

### Knockdown of CFL2 inhibits cell migration and invasion

**Figure 6:**
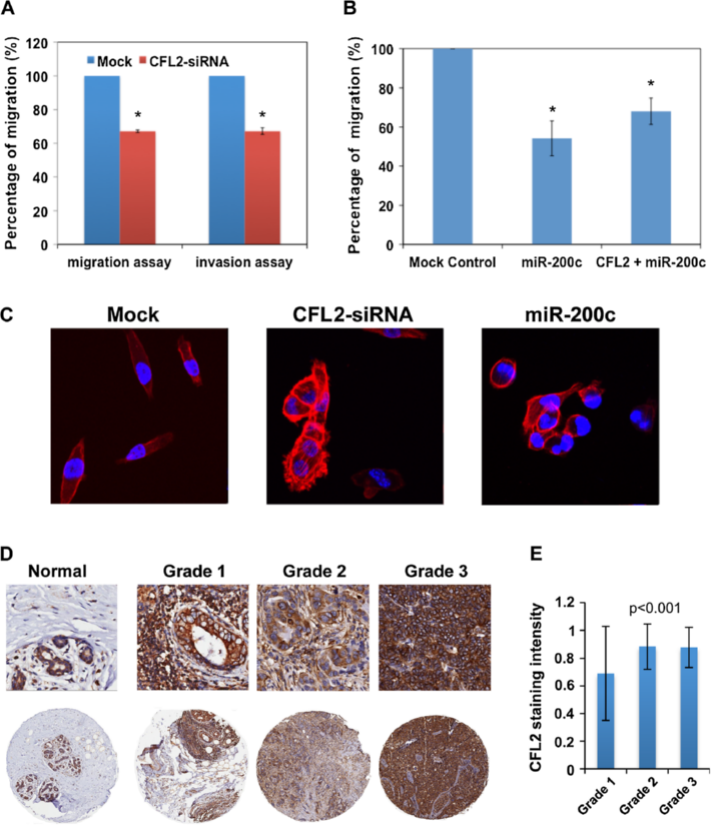
**CFL2 and breast cancer cell migration.** (**A**) Bar graph representing the normalized number of migratory or invaded cells after the respective siRNA transfection. (**B**) Overexpression of CFL2 can compensate the effect of exogenous miR-200c. Bar graph represents the normalized number of migratory cells after the respective transfection of miRNA mimic control, miR-200c mimic, and co-transfection of miR-200c and a plasmid encoding CFL2, respectively. *: Student’s *T*-test, p < 0.05. (**C**). Rhodamin-phalloidin staining of F-actin in the CFL2-siRNA and miR-200c transfected MDA-MB-231 cells. (**D**) Immunohistochemical analysis of TMA of primary breast tumors using antibodies against CFL2. Representative images of TMA cores are presented showing normal breast tissue, grades I, II, and III breast tumors with increasing staining intensities. (**E**) Bar graph representing the quantification of CFL2 immunostaining from blindly scored tissue microarray sections. Staining intensity of each sample was given a modified histochemical score (MH-score) that considers both the intensity and the percentage of cells stained at each intensity level. The intensity of each grade is the average of MH-score of all samples in that grade. Data were analyzed by one-way ANOVA with p-values as noted in the figure.

*CFL2*, which encodes cofilin-2, was significantly down regulated by miR-200c (Figures 4C and 5C). In addition, transfection of a miR-200c inhibitor in miR-200c-expressing cell lines, MCF7 and ZR-75-1 up-regulated CFL2 expression (Additional file 6: Figure S3). In order to further investigate the functional role of cofilin-2 in the migration and invasion of breast cancer cells, the MDA-MB-231 cells were transiently transfected with a *CFL2*-specific siRNA. Knockdown of *CFL2* led to inhibition of both migration and invasion by approximately 40% (Figures 6A). To further elucidate the specific role of miR-200c in regulating breast cancer cell migration through *CFL2* regulation, we co-transfected a miR-200c mimic with a plasmid encoded with *CFL2* cDNA in MDA-MB-231 cells. 48 hours after co-transfection, the MDA-MB-231 cells transfected with miR-200c mimic alone or co-transfected along with *CFL2* cDNA were analyzed for changes in cell migration. The *CFL2* cDNA does not contain the 3-UTR of endogenous *CFL2*, and therefore was not affected by miR-200c. As shown in Figure 6B, overexpression of *CFL2* partially reversed the effect of miR-200c on cell migration. These results indicate that miR-200c inhibited the migration of MDA-MB-231 through specific down-regulation of *CFL2,* and overexpression of *CFL2* can compensate the negative effect of miR-200c on cell migration. Because one of the functions of the cofilin gene family is to regulate F-actin turn over, we performed F-actin staining to examine the effect of *CFL2* knockdown on MDA-MB-231 cells. The depletion of cofilin-2 from MDA-MB-231 cells significantly increased F-actin levels (Figure 6C). Similarly, the miR-200c transfected MDA-MB-231 cells also displayed an increased level of F-actin staining although it was not as strong as in cells transfected with *CFL2* siRNA. Most *CFL2* knockdown cells were also larger relative to the wild-type cells. The increase in cell size may partly result from a flattened cell shape.

### CFL2 expression in primary breast cancer patient samples positively correlated with tumor grades

To determine if CFL2 is associated with breast tumor progression, we performed TMA analysis on 205 primary breast tumor and 5 normal breast tissue samples. The CFL2 expression can be seen to increase dramatically along with the grade of the tumor (p = 0.001, Figures 6D-E). The increase in CFL2 expression likely assists the higher-grade tumor cells in obtaining their migratory propensity by allowing for considerably higher F-actin turnover.

## Discussion

The complications arising from metastasis are the major causes of death from cancer. Mounting evidence suggests that miRNAs may promote or suppress tumor metastasis [12], thus offering a new perspective on the metastatic process. However, there has not been a systematic evaluation of the role of each miRNA, or a combination of coordinately expressed miRNAs, in the metastatic process of breast cancer cells. In this study, we attempt to perform a systematic evaluation of the role of miRNAs in the invasiveness of breast cancer cells using a group of 12 well-characterized breast cancer cell lines. Previous gene expression studies have shown that these cell lines resemble the primary tumor cells and can be grouped as basal or luminal-like breast cancer subtypes [17]. These cell lines are highly variable in their migration and invasion capability; BT474, MDA-MB-468, T47D, ZR-75-1, MCF7, SK-BR3 represent less-invasive breast cancer cell lines, while MDA-MB-231, HS578T, BT549, SUM159 are more invasive. MCF10A and MCF12A are two immortalized breast epithelial cell lines that are non-tumorigenic, but were classified as basal-like cell lines based on gene expression profiles. Using highly stringent criteria, we identified a group of 11 miRNAs that were most differentially expressed between the invasive and less-invasive cell lines, including several miRNAs that have been linked to breast cancer previously, such as miR-200c, miR-203, miR-205, miR-375, miR-141, and miR-146a [15,16,18]. Introduction of exogenous miR-200c, miR-205, and miR-375 mimics in the invasive breast cancer cell line MDA-MB-231 inhibited cell migration and invasion suggesting that these miRNAs may play important roles in maintaining the invasiveness of this cell line.

Using gene expression array analysis, we also identified candidate genes affected by the exogenous miRNA mimics in MDA-MB-231 cells. IPA analysis revealed that genes affected by miR-200c, miR-205, and miR-375 are involved in cellular movement and cell-to-cell signaling. Most significantly, our integrated analysis suggested that miR-200c might play a pivotal role in regulating cell migration and invasion, as 27 of the 35 differentially expressed genes were miR-200c targets. This is consistent with the migration and invasion experimental results showing that the miR-200c mimic is more potent than miR-205 and miR-375 mimics in inhibiting the migration and invasion of MDA-MB-231 cells. CDH1 (E-cadherin) was found to be at the core of the molecular network regulated by miR-200c. CDH1 acts as a hub connected by several neighborhood genes that play important roles in cell migration and invasion (Additional file 6: Figure S2A).

To further understand the function of the three miRNAs, it was necessary to identify their true targets in breast cancer cells. The computational prediction of miRNA targets still faces significant challenges. The most widely used tools (miRanda, TargetScan, PicTar, PITA, and RNAhybrid) are characterized by a significant proportion of false-positive interactions because post-transcriptional regulation is context-dependent. On the basis of increasing experimental evidence supporting the hypothesis that miRNAs can act through target degradation, it has been proposed that target predictions could be integrated with miRNA and gene expression profiles to select functional miRNA-mRNA relationships [19-24]. In this study, we performed an integrated analysis by combining multiple data sets: 1) differentially expressed miRNA between invasive and less invasive cell lines; 2) differentially expressed genes between the two groups of cell lines; 3) miRNA target genes predicted by TargetScan; 4) genes significantly down-regulated upon forced expression of a miRNA. The integrated analysis yielded 35 genes that satisfied the selection criteria. Out of the 27 differentially expressed target genes identified for miR-200c, 17 have been shown to influence the migration, invasion, or metastasis of cancer cells both *in vitro* and *in vivo* (Table 1). Of these genes, *LOX*, which encodes Lysyl oxidase, is essential for hypoxia-induced metastasis [25] and it was reported that breast cancer cell metastasis can be attenuated by lysyl oxidase inhibitors [26]. EMP1 may represent a novel immunohistochemical marker helpful in distinguishing between invasive ductal and lobular carcinomas [27]. Fascin (*FSCN1*) is a key regulator of breast cancer invasion [28]. CDH11 may play a role in recruiting Trio to the plasma membrane where Trio activates Rac, leading to cell migration [29]. *SEC23A*, which mediates the secretion of metastasis-suppressive proteins including *IGFBP4*, has been shown to be a direct target of miR-200c, and the subsequent down-regulation has been correlated to an increase in metastatic colonization [30]. Interestingly, *SEC23A* was identified as a target of both miR-200c and miR-375 (Table 1), suggesting crosstalk can occur between these two miRNAs. *PRKCA* overexpression is strongly associated with a more invasive and metastatic phenotype in breast cancer [31]. Furthermore, it has been shown that *PRKCA* expression is increased in the invasive breast cancer cell lines MDA-MB-231 and HS578T, and overexpression can lead to a significant increase in both the migration and invasion ability of the cell lines [32]. Finally, SLC7A11, which is functional subunit of the cystine/ glutamate transporter, plays an important role in breast tumor metastasis and maybe a potential target for cancer therapy [33].

Among the target genes identified, *CFL2* was most significantly down-regulated by miR-200c. Hurteau et al. first indicated that CFL2 as a potential target gene of miR-200c [30], and later re-reported by Gregory et al [34]. Korpal et al showed recently that knockdown of *Cfl2* in a mouse mammary tumor cell lines 4TO7 significantly decreased cell migration [35]. In this study, we further revealed that *CFL2* plays a crucial role in regulating actin turnover and is intimately linked to the cell migration and invasion ability. An increased level of CFL2 would allow for a much higher F-actin turnover rate, thus allowing the cell to become much more mobile [36]. Therefore by slowing the F-actin turnover rate, miR-200c helps to maintain the anchoring filaments that tend to hold the cells in place, leading to the decreased migration and invasion ability. CFL2 expression was also noted to increase significantly along with the grade of the tumor. This increase in CFL2 production likely assists the tumor in spreading both locally and metastatically at a much greater rate.

In summary, we have performed the first systematic screening of miRNA-mRNA target pairs that are differentially expressed between invasive and less-invasive breast cancer cells. We identified a group of negatively correlated miRNA-mRNA target pairs via integrated analysis of miRNA and mRNA expression profiles. The subsequent confirmation studies demonstrated that this integrated approach is very effective. Our results further emphasize the important role of miR-200c and its target genes in maintaining the invasiveness of breast cancer cells. Further analysis of the candidate miRNAs and their target genes identified in this study may ultimately lead to the identification of novel prognostic biomarkers and therapeutic targets.

## Abbreviations

3′-UTRs: 3^′^-untranslated regions; ANOVA: Analysis of variance; FBS: Fetal bovine serum; IPA: Ingenuity Pathway analysis; miRNA: microRNA; qRT-PCR: Quantitative reverse transcriptase polymerase chain reaction; TMA: Tissue microarray analysis.

## Competing interests

The authors declare that they have no competing interests.

## Authors’ contributions

DL, LH and HS designed the study. DL, JMW, NH, JL, and LP performed the experiments. DL, NH, LH and SH participated in the data analysis and interpretation. SH contributed vital reagents and analytical tools. DL, JMW and HS co-wrote the manuscript. All authors read and approved the final manuscript.

## Supplementary Material

Additional file 1: Table S1 List of primers used for qRT-PCR analysis. Click here for file

Additional file 2: Table S2 List of primers used to amplify 3^′^-UTR. Click here for file

Additional file 3: Table S3 Clinical characteristics of the primary breast tumor specimens analyzed in the TMA panel. Click here for file

Additional file 4: Table S4 List of genes up-regulated in aggressive cell lines vs. normal and less aggressive cell lines. Click here for file

Additional file 5: Table S5 List of genes down-regulated in aggressive cell lines vs. normal and less aggressive cell lines. Click here for file

Additional file 6: Figure S1 Ingenuity Pathways Analysis of the differentially expressed genes in the miR-200c (**A**), miR-205 (**B**), and miR-375 (**C**) mimics transfected MDA-MB-231 cells revealed the enrichment of different functional groups respectively. **Figure S2.** Ingenuity Knowledge Base Analysis of the differentially expressed genes in the miR-200c (**A**), miR-205 (**B**), and miR-375 (**C**) mimics transfected MDAMB- 231 cells identified interesting molecular interaction networks. **Figure S3.** qRT-PCR analysis of CFL2 expression in miR-200c inhibitor transfected MCF7 and ZR-75-1 cell lines that express endogenous miR-200c. miR-200c inhibitor (Ambion, Austin TX) was transfected at a concentration of 50nM with Lipofectmine 2000 (Invitrogen). Total RNA was harvested at 48 hr after transfection and converted to cDNA for qRT-PCR analysis. Click here for file

Additional file 7: Table S6 List of genes down-regulated in miR-200c mimic transduced MDA-MB-231 cells. Click here for file

Additional file 8: Table S7 List of genes down-regulated in miR-205 mimic transduced MDA-MB-231 cells. Click here for file

Additional file 9: Table S8 List of genes down-regulated in miR-375 mimic transduced MDA-MB-231 cells. Click here for file
