# Supplementary material for: A systematic evaluation of miRNA:mRNA interactions involved in the migration and invasion of breast cancer cells
Source: J Transl Med. 2013 Mar 5;11:57. doi: 10.1186/1479-5876-11-57 (PMC3599769; doi:10.1186/1479-5876-11-57)
Supplement: Additional file 1: Table S1 — List of primers used for qRT-PCR analysis. [file 1479-5876-11-57-S1.docx]

**Supplemental Table 1.** List of primers used for qRT-PCR analysis.

| *Name* | *Forward* | *Reverse* |
| --- | --- | --- |
| β-actin | 5’-ATTGGCAATGAGCGCTTC-3’ | 5’-TGAAGGTAGTTTCGTGGATGC-3’ |
| ZEB1 | 5’-TGTTACCAGGGAGGAGCAGT-3’ | 5’-TGCCCTTCCTTTCCTGTGT-3’ |
| CFL2 | 5’-AGTGCCACAGAGCCGAAG-3’ | 5’-TCATCATTCACTGTAACTCCAGAAG-3’ |
| Vimentin | 5’-TGAGATTGCCACCTACAGGA-3’ | 5’-GAGGGAGTGAATCCAGATTAGTTT-3’ |
| CDH11 | 5’-TTTCGGTGGAAGCACAGAC-3’ | 5’-CTTGGCCTGGATCACCAC-3’ |
| LAMC1 | 5’-GTGCTGTTGTTCCCAAGACA-3 | 5’-GCCATCATCACAGAGCTCAC-3’ |
| PRKCA | 5’-GTGCTGTTGTTCCCAAGACA-3’ | 5’-CTTGAATGGTGGCTGGATCT-3’ |
| SEC23A | 5’-AGTCAACCCTTCACAGACTCATAA-3’ | 5’-TCATCTGTAAGAATAGGTGCTCCA-3’ |
| TIMP2 | 5’-GAAGAGCCTGAACCACAGGT-3’ | 5’-CGGGGAGGAGATGTAGCAC-3’ |
| PTPRJ | 5’-CTCGGACAGTTTGCAATAGAACT-3’ | 5’-ACTCGGAAGCACCGTCAG-3’ |
| PTPRM | 5’-CAGTTTGCTACCTTCCAGTGC-3’ | 5’-AGAGGAGCATCTCGCACATC-3’ |
| LDHB | 5’-GATGGATTTTGGGGGAACAT-3’ | 5’-CTGCCACATTCACACCACTC-3’ |
