# Supplementary material for: A systematic evaluation of miRNA:mRNA interactions involved in the migration and invasion of breast cancer cells
Source: J Transl Med. 2013 Mar 5;11:57. doi: 10.1186/1479-5876-11-57 (PMC3599769; doi:10.1186/1479-5876-11-57)
Supplement: Additional file 2: Table S2 — List of primers used to amplify 3′-UTR. [file 1479-5876-11-57-S2.docx]

**Supplemental Table 2. List of primers used to amplify 3’-UTR.**

| *Name* | *Forward* | *Rverse* |
| --- | --- | --- |
| CDH11 | 5’-CTAGCTAGCcaataacgatacaaatttggcc-3’; | 5’-acgcgtcgacttaaaaaaaaatctttttttatttcaaag-3’ |
| CFL2 | 5’-CCGCTCGAGAATGACAGTCAAGTGCCATCTGG-3’ | 5’-TGCTCTAGAGAAATACCAGTTTTTAAACTTTTAATG-3’ |
| SEC23A | 5’-CTAGCTAGCagtgctaataatgttaaagacac-3’ | 5’-acgcgtcgacaacagtgaaaagtatattttattggc-3’ |
| ZEB1 | 5’-CTAGCTAGCtcgtttttctagaaggaaaataaattc-3’ | 5’-acgcgtcgacAAGGCAATAGAAAAAGAAGGC-3’ |
| PTPRM | 5’-CTAGCTAGCtggtgtaaacagctctgcaaac-3’ | 5’-acgcgtcgacgtgcttcacaaaaatttcttttaatg-3’ |
| LDHB | 5’-CTAGCTAGCCTAGTGAGCTCTAGGCTGTAG-3’ | 5’-acgcgtcgacGCACACTACAATAGTTAATTTTA-3’ |
