# Supplementary material for: A systematic evaluation of miRNA:mRNA interactions involved in the migration and invasion of breast cancer cells
Source: J Transl Med. 2013 Mar 5;11:57. doi: 10.1186/1479-5876-11-57 (PMC3599769; doi:10.1186/1479-5876-11-57)
Supplement: Additional file 6: Figure S1 — Ingenuity Pathways Analysis of the differentially expressed genes in the miR-200c (A), miR-205 (B), and miR-375 (C) mimics transfected MDA-MB-231 cells revealed the enrichment of different functional groups respectively. Figure S2. Ingenuity Knowledge Base Analysis of the differentially expressed genes in the miR-200c (A), miR-205 (B), and miR-375 (C) mimics transfected MDAMB- 231 cells identified interesting molecular interaction networks. Figure S3. qRT-PCR analysis of CFL2 expression in miR-200c inhibitor transfected MCF7 and ZR-75-1 cell lines that express endogenous miR-200c. miR-200c inhibitor (Ambion, Austin TX) was transfected at a concentration of 50nM with Lipofectmine 2000 (Invitrogen). Total RNA was harvested at 48 hr after transfection and converted to cDNA for qRT-PCR analysis. [file 1479-5876-11-57-S6.pdf]

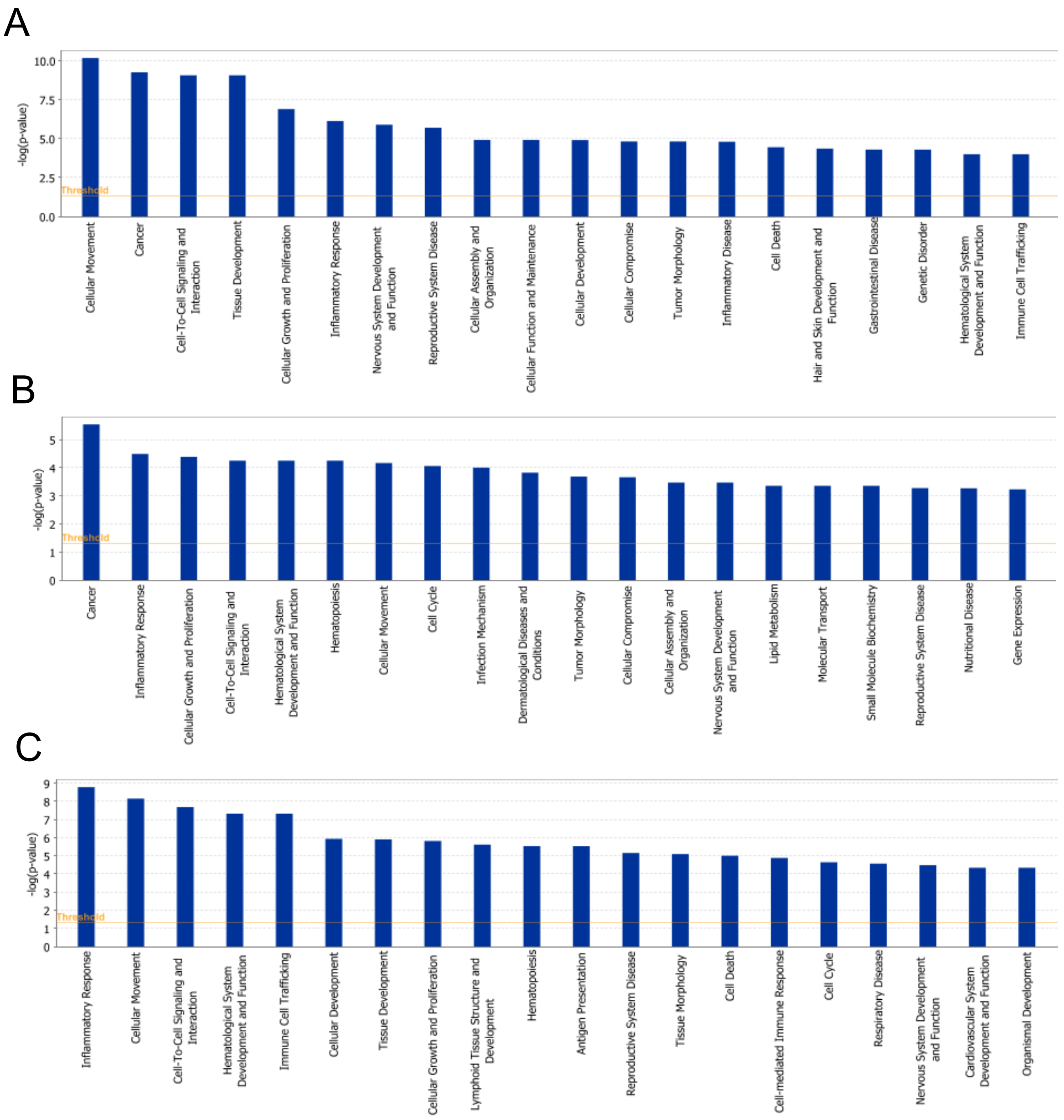

Supplementary Figure 1. Ingenuity Pathways Analysis of the differentially expressed genes in the miR-200c (**A**), miR-205 (**B**, and miR-375 (**C**) mimics transfected MDA-MB-231 cells revealed the enrichment of different functional groups respectively.

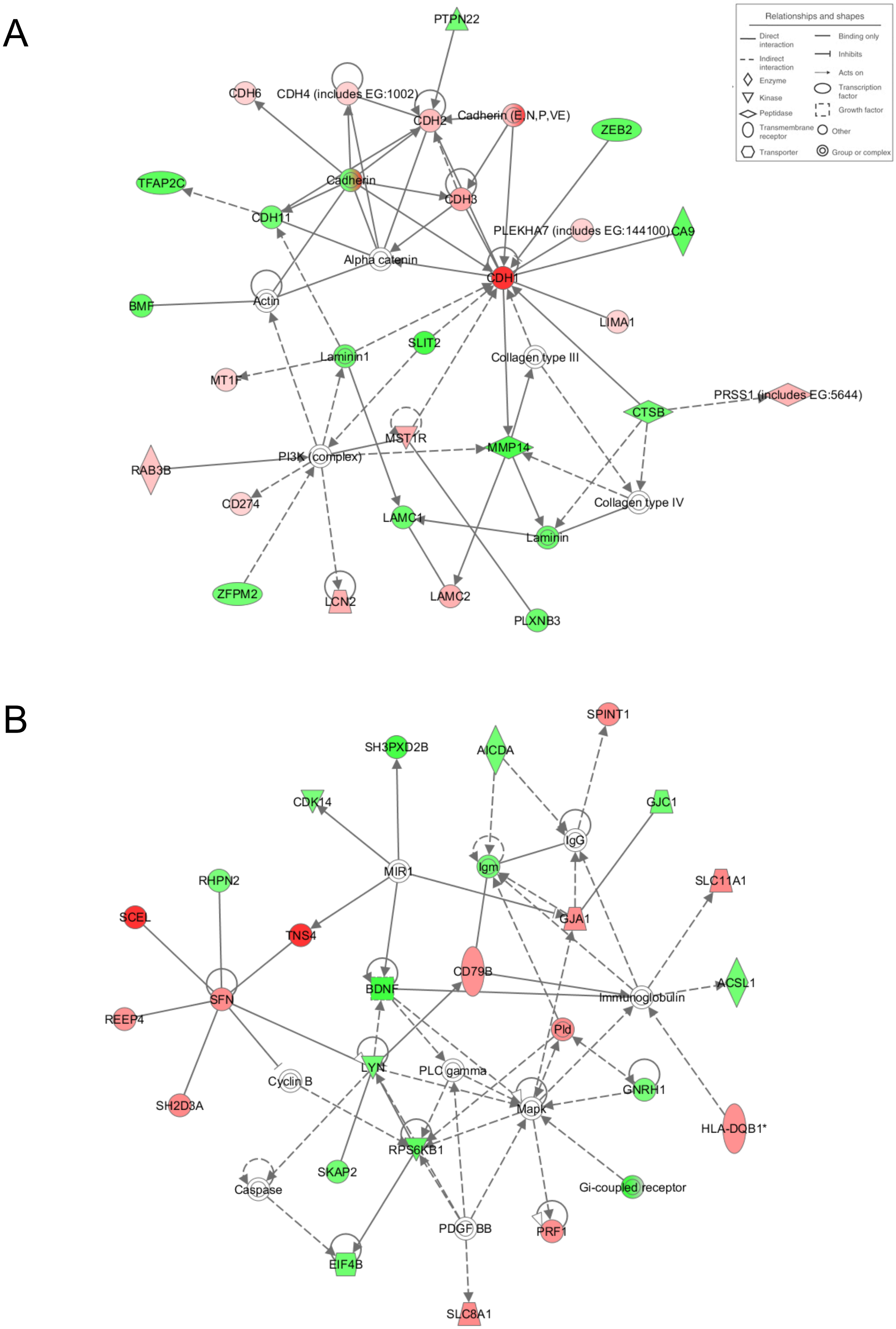
A
Relationships and shapes
Direct interaction
Indirect interaction
Enzyme
Kinase
Peptidase
Transmembrane receptor
Transporter
Binding only
Inhibits
Acts on
Transcription factor
Growth factor
Other
Group or complex
PTPN22
CDH6
CDH4 (includes EG:1002)
CDH2
Cadherin (E,N,P,VE)
ZEB2
TFAP2C
Cadherin
CDH3
CDH11
PLEKHA7 (includes EG:144100)
CA9
Alpha catenin
CDH1
BMF
Actin
LIMA1
SLIT2
Laminin1
Collagen type III
MT1F
PRSS1 (includes EG:5644)
CTSB
MST1R
RAB3B
PI3K (complex)
MMP14
CD274
Collagen type IV
LAMC1
Laminin
ZFPM2
LCN2
LAMC2
PLXNB3
B
SPINT1
SH3PXD2B
AICDA
CDK14
GJC1
IgG
Igm
MIR1
RHPN2
SLC11A1
SCEL
GJA1
TNS4
CD79B
ACSL1
BDNF
SFN
REEP4
Immunoglobulin
Pld
LYN
PLC gamma
Cyclin B
GNRH1
SH2D3A
Mapk
HLA-DQB1*
RPS6KB1
SKAP2
Caspase
Gi-coupled receptor
PDGF BB
PRF1
EIF4B
SLC8A1

C

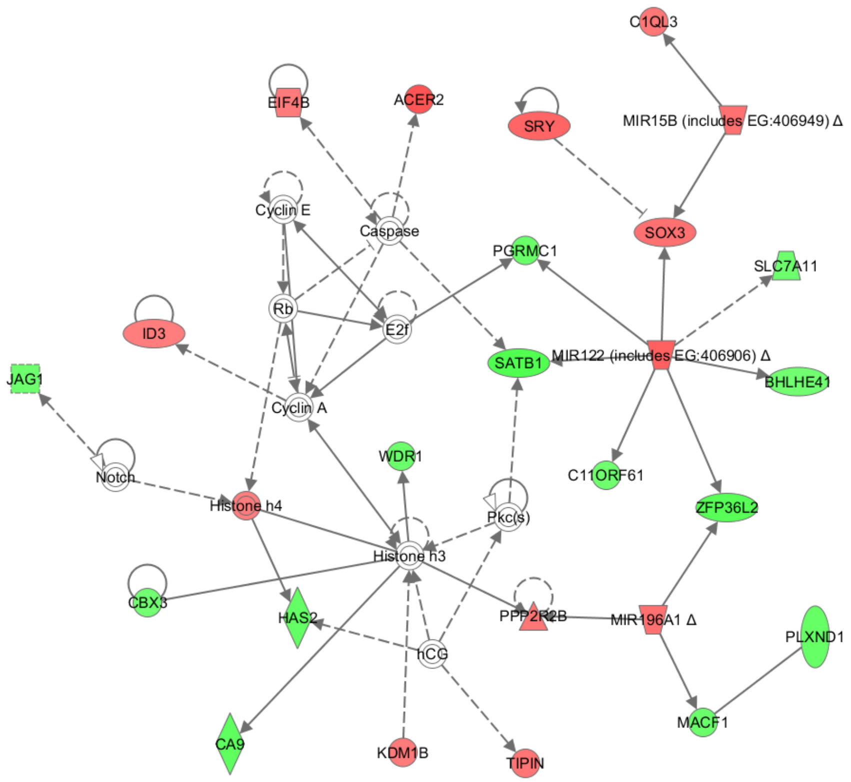

Supplementary Figure 2. Ingenuity Knowledge Base Analysis of the differentially expressed genes in the miR-200c (**A**), miR-205 (**B**), and miR-375 (**C**) mimics transfected MDA-MB-231 cells identified interesting molecular interaction networks.

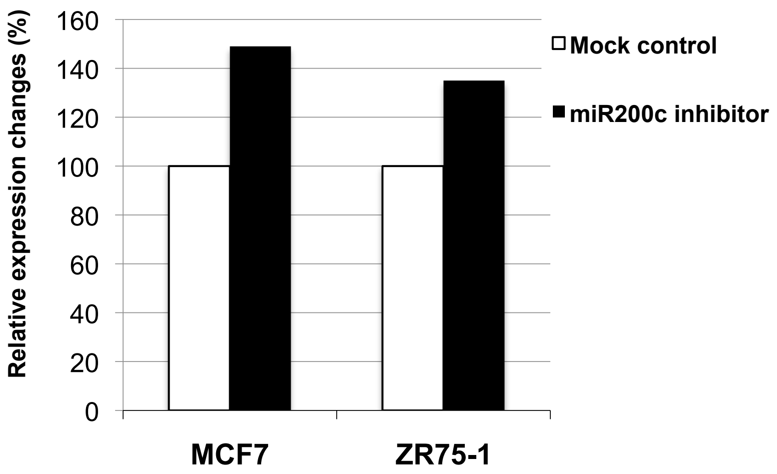

Supplementary Figure 3. qRT-PCR analysis of CFL2 expression in miR-200c inhibitor transfected MCF7 and ZR-75-1 cell lines that express endogenous miR-200c. miR-200c inhibitor (Ambion, Austin TX) was transfected at a concentration of 50nM with Lipofectmine 2000 (Invitrogen). Total RNA was harvested at 48 hr after transfection and converted to cDNA for qRT-PCR analysis.
